# Grain Selection in a High-Efficiency 2D Grain Selector During Casting of Single-Crystal Superalloys

**DOI:** 10.3390/ma12050789

**Published:** 2019-03-07

**Authors:** Xintao Zhu, Fu Wang, Dexin Ma, Andreas Bührig-Polaczek

**Affiliations:** 1 Foundry Institute, RWTH Aachen University, Intzestrasse 5, 52072 Aachen, Germany; zhudb8@gmail.com (X.Z.); d.ma@gi.rwth-aachen.de (D.M.); sekretariat@gi.rwth-aachen.de (A.B.-P.); 2 State Key Laboratory for Manufacturing System Engineering, School of Mechanical Engineering, Xi’an Jiaotong University, Xi’an 710049, China

**Keywords:** grain selection, directional solidification, single crystal, Ni-based single crystal superalloys

## Abstract

Using electron backscattered diffraction techniques (EBSD) and optical microscopy (OM), the grain selection and competitive growth in a new-designed high-efficiency two-dimensional (2D) selector during solidification of Ni-based single-crystal (SX) superalloys have been investigated with emphasis on the geometry of the selector part in this article. It is found that the efficiency of the grain selector depends greatly on the thickness and eccentric distance of the selector part. When the thickness is smaller than 3 mm, a single grain can be selected. After reducing this value, the grain selector becomes more effective. When the eccentric distance is larger than 8 mm, one grain can be selected. As the eccentric distance increases, the selector’s efficiency is optimized. Recommendations for optimizing the geometry of the selector part are provided.

## 1. Introduction

Owing to the elimination of grain boundaries (GB), single crystal (SX) blades of Ni-based superalloys exhibit excellent high-temperature performances, have been widely used in aero-engines and are being introduced into industrial gas turbines (IGTs) [1,2,3]. In comparison with equiaxed (EQ) and directional solidified (DS) blades, the preferred <001> crystallographic orientation of SX blades of Ni-based superalloys coinciding with the minimum in Young’s modulus is oriented parallel to the blades’ axis [4]. For this reason, the creep performance can be optimized as long as the axis direction of SX blades approaches the <001> orientation. Nevertheless, a slight deviation from the <001> orientation can lead to a great reduction in the creep property of SX blades [5]. Consequently, the direction of the <001> orientation close to the axial direction of SX blades is crucial for obtaining high-temperature performance of SX blades. To achieve single-crystal structure, grain selectors are conventionally used to ensure that one grain is selected and can grow into a SX blade during directional solidification [6]. A grain selector generally consists of a starter block, a selector part and a connector part. Prior studies [7,8,9,10,11,12,13] suggest that the geometries of the starter block and the selector part of grain selectors in particular can affect significantly grain selecting efficiency and the crystallographic orientation of the selected grain. Dai et al. [8,11,13] have studied the microstructure evolution and grain competitive growth in spiral grain selector and suggested that the spiral selector becomes more efficient in grain selection with a smaller spiral thickness (dT), a larger spiral diameter (dS) and smaller takeoff angle. Meng et al. [9,10] have placed the grain selection process in a spiral selector systematically and proposed that the geometry of the grain selector (the height of starter block, the spiral diameter and initial angle) plays an important role in grain selection. The dimension of selector should be maintained in a stable range to optimize the grain orientation and select a single crystal efficiently.

To achieve high-efficiency grain selectors for growing SX blades, several kinds of grain selectors such as restrictor, angled and spiral selectors were designed in the past few years [14]. However, the restrictor selector was less efficient because a longer length for selecting a single grain is needed [15]. Around the corners of the angled selector, new grains often nucleate due to the sudden change in growth direction [16,17]. In the wax injection stage, the spiral selector cannot be integrally fabricated coupled with the blades, because of the complex, three-dimensional (3D) shape of the spiral. It needs to be welded with the blades, which results in inevitably some position error and sudden failure in the grain selection. A high stability of the spiral selector cannot be kept, although it has been employed in casting foundries.

**Figure 1:**
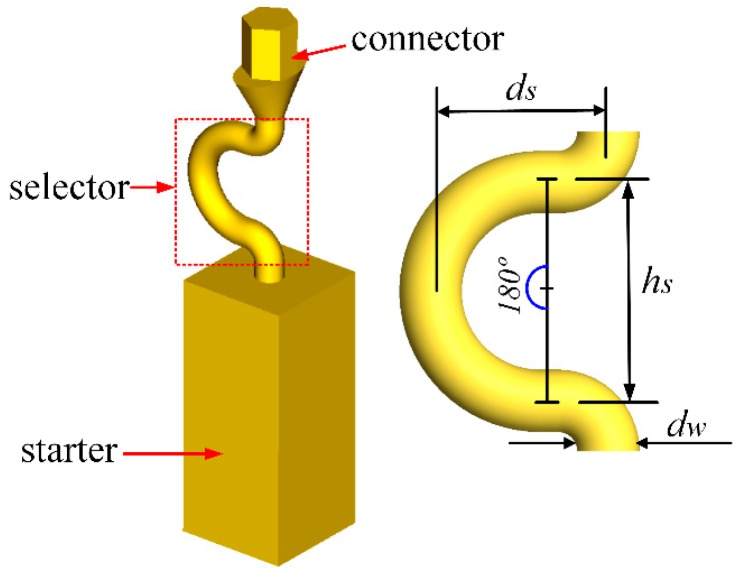
Schematic drawings of the new 2D grain selector and its selector part showing parameters used in the selector part.

To overcome these shortcomings, a new 2D grain selector was designed in this study. H. Esaka et al. have developed a mathematical mode of the 2D grain selector [18]. Moreover, H. Gheisari and E. Karamian have studied the dendritic growth mechanism in a 2D Zigzag grain selector [19]. A series of industrial Bridgman-experiments were conducted in foundry institute of RWTH Aachen University to exhibit the physical processes occurring in the selector parts with different selector’s thickness (*d_w_*) and eccentric distance (*d_s_*), whereas the height (*h_s_*) and curved angle (*θ*) were kept constant, as shown in Figure 1. The effect of the geometry of the selector part on grain selection was quantified by using electron backscatter diffraction (EBSD) equipped in scanning electronic microscopy (SEM) and optical microscopy (OM). Based on the results, the influences of the selector’s thickness and eccentric distance are discussed, and a mechanism accounting for the experimental results is put forward.

## 2. Experiments

### 2.1. Model Design of 2D Grain Selector

As illustrated in Figure 1, the 2D selector consists of three parts: a starter block, a C-form selector part and a connector part. In this study, the starter block was designed in quadrangle of 10 mm (L) × 10 mm (W) × 30 mm (H). The selector parts were designed with fixing height (*h_s_*) and curved angle (*θ* = 180°) but different routes of grain selection by varying thickness (*d_w_* = 2.6~4.2 mm) and eccentric distance (*d_s_* = 6~20 mm), as listed in Table 1 and Table 2. Due to the low strength of the selector with the thickness smaller than 2.6 mm, the wax 2D selector is easy to deform during assembling with wax castings to wax cluster, and these selectors are not investigated.

### 2.2. Directional Solidification Experiments

The superalloy CM247LC was used in this investigation. The chemical composition of CM247LC is listed in Table 3.

**Figure 2:**
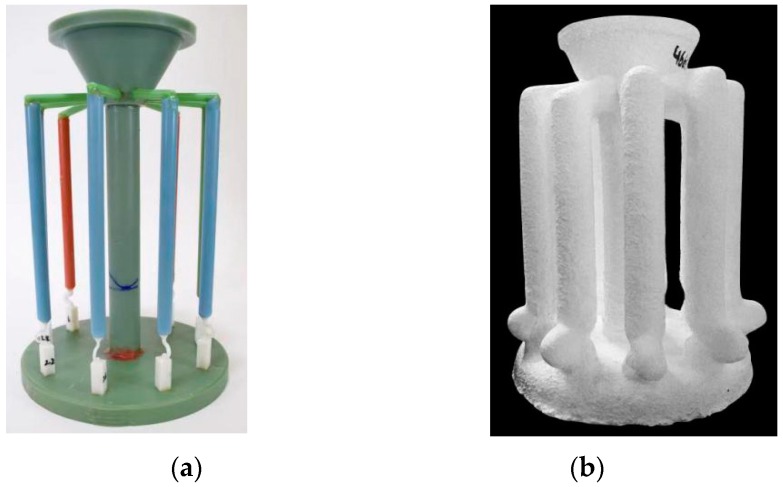
Images of the wax model (**a**) and the shell mold (**b**).

To investigate the effect of selector geometry on grain selection, the wax units consisting of 2D selectors with different size and ∅20 mm × 150 mm cylindrical bars were injected integrally. Ten units were assembled around a central rod to form a wax mold cluster, as shown in Figure 2a. The wax assembles were then dipped into a water-based ceramic slurries having different viscosities, and stuccoed using different sizes of alumina sand. The dipping and stuccoing operations were repeated until the wall-thickness of the shell mold cluster reached 7~8 mm. After drying, the mold cluster was dewaxed in a steam autoclave. Subsequently, the mold was sintered to remove the remaining wax and increase its strength. Finally, the shell mold (shown in Figure 2b) was mounted on a water-cooled copper chill in a Bridgman furnace (ALD vacuum Technologies, VIM-5, Foundry institute, RWTH Aachen University, Aachen, Germany). A withdrawal rate of 3 mm/min was used in these experiments. After heating and withdrawal process, the mold was cooled in the cooling zone of the furnace. When the temperature of the heater decreased below 300 °C, the vacuum was released and the casting mold was removed. The entire casting cluster was then knocked out of the ceramic mold and the units were appropriately separated from the cluster.

### 2.3. Microstructural Characterization

The samples were sand blasted to remove any ceramic debris attached to their surface. After cleaning the sample, the selectors were cut and polished for microstructural analyses. 60 mL C_2_H_5_OH + 40 mL HCl + 2g Cu_2_Cl∙2H_2_O etchant was employed to reveal the microstructures and the EBSD method was employed for the orientation.

## 3. Results and Discussion

### 3.1. Grain Structure Evolution in the Selector Part of 2D Selectors

**Figure 3:**
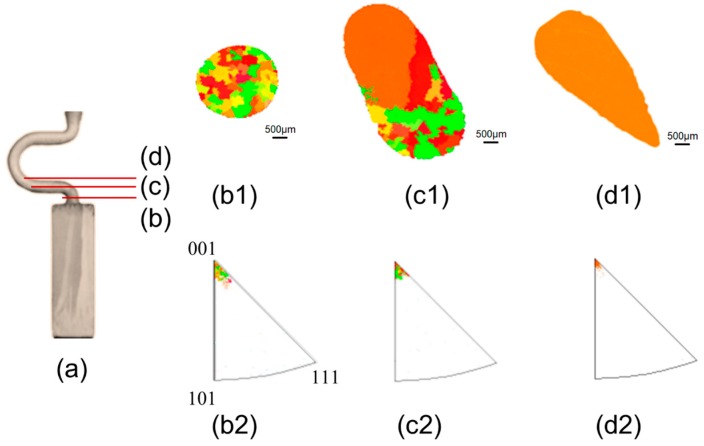
Optical image of the selector part of the 2D grain selector having 3 mm thickness and 8 mm eccentric distance (**a**); EBSD IPF-X maps on the grain structure evolution (**b1,c1,d1**) and the EBSD inverse pole figures in growth direction at different heights (**b2,c2,d2**).

Figure 3 shows the grain structure evolution in the selector part of the 2D selector with selector thickness of 3 mm and eccentric distance of 8 mm, as a typical example. Figure 3a illustrates the macrostructure of the selector. Figure 3(b1,c1,d1) exhibit the grain structure at transversal sections corresponding to different heights along the selector (shown in Figure 3a), which was analyzed by using EBSD method. Figure 3(b2,c2,d2) show the corresponding <001> inverse pole figures (IPFs) which reveals the orientation of the grains. It is found that with increasing height the quantity of the grains decreased significantly, but the grain enlarged gradually (shown in Figure 3(b1,c1,d1)). At the height of approximately 37 mm, a single crystal was selected. A major reduction in the number of the grains in the selector part of the 2D selector demonstrated the high-efficiency in grain selection. 

### 3.2. Effect of Selector Thickness on Grain Selection

**Figure 4:**
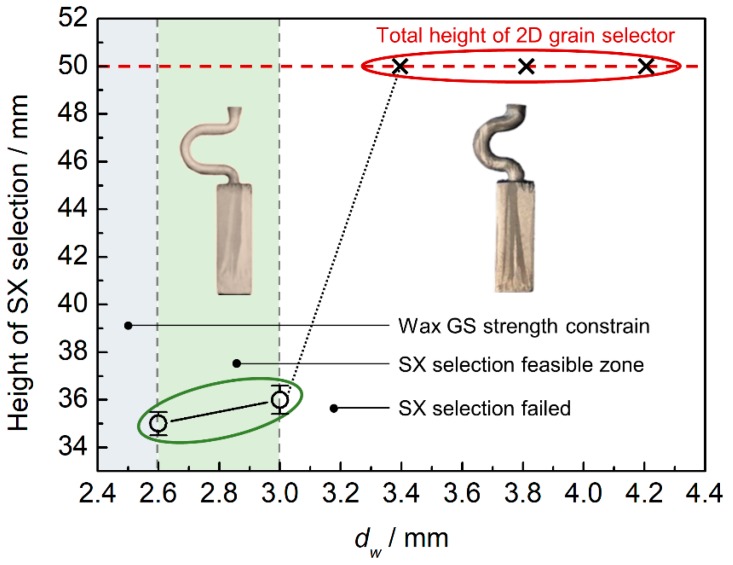
Experimentally observed heights of SX position versus selector thickness (*d_w_*).

Figure 4 shows the effect of the selector thickness on grain selection. The eccentric distance is kept as 8 mm. It can be seen that with increasing selector thickness to from 2.6 mm to 3 mm, a single crystal can be selected and the height where the SX structure occurs is reduced gradually. However, the SX selection failed when the thickness is larger than 3 mm. Also considering the stability of the 2D selector, the thickness of 3 mm is recommended.

**Figure 5:**
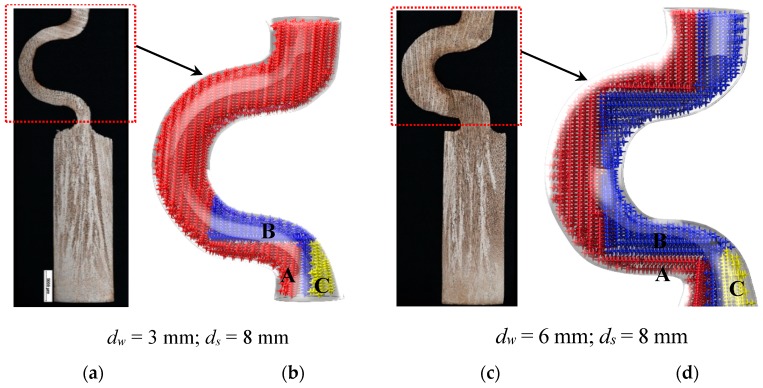
Optical images of the dendrite structure in the selector parts of the selectors with 3 mm (**a**) and 6 mm (**c**) thickness as well as their corresponding schematic diagrams (**b**) and (**d**).

Previous investigations [16,20] suggests that grain selection in the selector part arose from the dendrite competitive growth and geometrical blocking mechanism. As shown in Figure 5a,b, three grains entered into the selector part of the 2D selectors with 3 mm and 6 mm diameters. The orientation of grain B is well aligned with the vertical thermal gradient, whereas the orientations of grains A and C are a little misaligned. At the beginning of solidification, the dendrite tips of grain B can grow ahead of grains A and C because of the lower undercooling demanded at the dendrite tips for grain B [19]. The grain C is surpassed by grain B since its primary tips impinge not only on the mold wall but also on the dendrite trunks of grain B. Although grain B grows ahead, it is finally surpassed by grain A. The first reason for this occurrence is that the primary and tertiary dendrite arms of grain B are blocked by the selector wall. The second reason is that the growing velocity of the secondary dendrite arms that develops from the primary dendrite arms of grain B is slower than for grain A, because of the fine cooling condition and higher undercooling on the side facing the heater [21]. The tertiary dendrite arms of grain A blocks the growth of those of grain B. As a result, only grain A survives and grows into the castings. Figure 5c,d demonstrate the case when the selector thickness is increased. In this case, the grain B has enough available space for growing dendrites, which overcomes the shortage of the dendrite growing velocity. Therefore, both grain B and grain A can grow into the castings.

### 3.3. Effect of Eccentric Distance on Grain Selection

**Figure 6:**
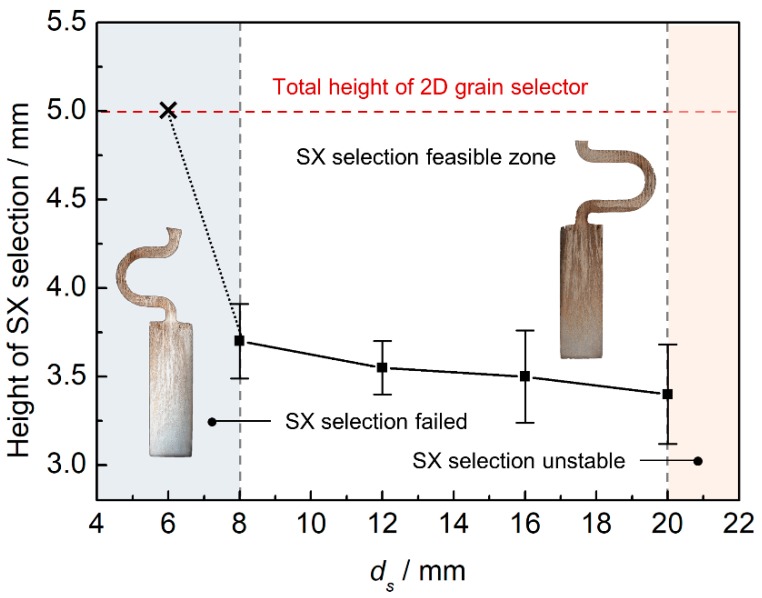
The relationship between heights of SX position and selector eccentric distance (*d_s_*).

Figure 6 illustrates the effect of eccentric distance on grain selection. The selector thickness is held constant as 3 mm. It is found that the SX can be successfully selected when the eccentric distance (*d_s_*) is larger than 8 mm. The selecting height is reducing with an increasing eccentric distance. This indicates that the selecting efficiency of the 2D selectors is improved with increasing eccentric distance. However, when the eccentric distance is larger than 20 mm, the structural instability occurs. The wax 2D selectors are easy to break when they are assembled with castings to a mold cluster. Considering the stability of the 2D selector, the eccentric distance of 8 mm is recommended.

**Figure 7:**
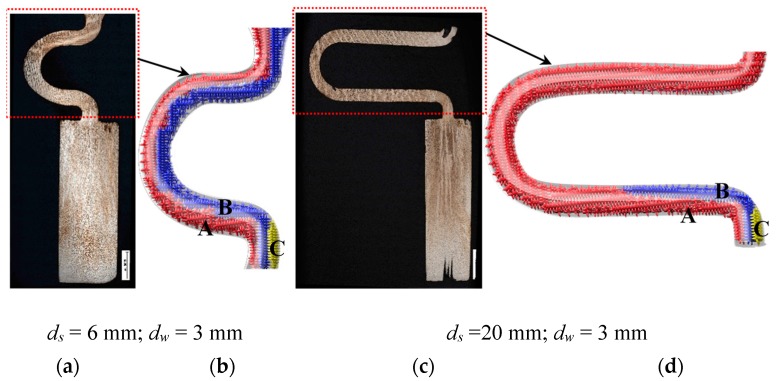
Optical images of the dendrite structure in the selector parts of the selectors with 3 mm (**a**) and 20 mm (**c**) eccentric distance as well as their corresponding schematic diagrams (**b**) and (**d**).

Figure 7 shows the dendrite structure in selector parts of the selectors with 3 mm and 20 mm eccentric distance as well as their corresponding schematic diagrams. It can be seen that three grains grow from the starter block into the selector part of the 2D selectors. Due to the well alignment with the vertical thermal gradient and the reduced undercooling required as a result, the dendrite tips of grain B grow ahead of the dendrite tips of grains A and C. Similarly to the case for selector thickness, the grain C is overgrown by grain B since its primary tips impinge not only on the mold wall but also on the dendrite trunks of grain B. When the selector’s eccentric distance is smaller, grains A and B can grow into the castings owing to having enough growing space or a larger undercooling. However, when the eccentric distance increases, the position of grain A achieves better cooling conditions due to the suspended structure. The growing and branching velocity of the dendrite arms of grain A is larger than that of grain B, which blocks the growing of grain B. Consequently, only grain A can survive and grow into the castings.

## 4. Conclusions 

By using optical microscopy and EBSD techniques, the grain selection in the selector part of a novel 2D grain selector was investigated experimentally with different geometrical parameters. The results obviously show the selection behavior of the selector part of the grain selector, and that smaller thickness and larger eccentric distance are more efficient in grain selection. Considering the stability of the selector part of the 2D grain selector, the selector’s thickness and eccentric distance of 3 mm and 8 mm, respectively, are recommended. It is recommended that grain selection in the selector part of the 2D grain selector during solidification is dominated by the geometrical blocking and the local thermal condition. Moreover, the results obtained from the 2D selector can provide a certain inspiration for future 3D grain selector design.

## Figures and Tables

**Table 1:** Variation in thickness of the selector part.

Case	1	2	3	4	5
*h_s_*/mm	constant	-	-	-	-
*θ*/deg	180°	-	-	-	-
*d_w_*/mm	2.6	3.0	3.4	3.8	4.2

**Table 2:** Variation in eccentric distance of the selector part.

Case	6	7	8	9	10
*h_s_*/mm	Constant	-	-	-	-
*θ*/deg	180°	-	-	-	-
*d_s_*/mm	6	8	12	16	20

**Table 3:** The composition of superalloy CM247LC/wt.%.

Elements	Al	Ti	Cr	Mo	Co	W	Ta	Hf	C	Ni
wt.%	5.49	0.74	8.03	0.5	9.41	9.87	2.9	1.36	0.094	Bal.
